# Age-Specific Rural-Urban Disparities in the Incidence of Ischemic Stroke in the Netherlands

**DOI:** 10.1212/WNL.0000000000210102

**Published:** 2024-11-25

**Authors:** Esmée Verburgt, Jamie I. Verhoeven, Nina A. Hilkens, Ilonca Vaartjes, Frank-Erik De Leeuw

**Affiliations:** From the Department of Neurology (E.V., J.I.V., N.A.H., F.-E.D.L.), Radboud University Medical Center, Donders Institute for Brain, Cognition and Behavior, Nijmegen; and Department of Epidemiology (I.V.), Julius Center for Health Sciences and Primary Care, University Medical Center Utrecht, the Netherlands.

## Abstract

**Background and Objectives:**

Multiple studies found a higher ischemic stroke incidence in rural areas compared with urbanized areas, often explained by a low socioeconomic status (SES). However, this has rarely been investigated specifically in younger adults. We aimed to investigate the age-specific (15–49 years vs 50+ years) incidence of ischemic stroke in rural and urbanized municipalities within the Netherlands.

**Methods:**

Patients with a first-ever ischemic stroke (15 years or older) between 1998 and 2018 were included in this registry-based study through linkage of Dutch national hospital administrative registries. Ischemic stroke was defined through *ICD-9* and *ICD-10* codes. The urbanization grade of the municipality was defined by the address density in 5 subgroups (from most urban ≥2,500 addresses per km^2^ to rural <500 addresses per km^2^). The urbanization grade-specific incidence rate per 100,000 person-years, standardized for age and sex, and incidence rate ratios (IRRs) were calculated. In addition, we performed stratified analyses for young age groups (15–39 and 40–49 years) and neighborhood SES (nSES), which was calculated using welfare, level of education, and recent labor participation.

**Results:**

In total, 23,720 patients aged 15–49 years (median age 44.7 years [interquartile range (IQR) 40.6–48.8], 51.6% women) and 369,107 patients aged older than 50 years (median age 76.7 years [IQR 68.8–84.7], 50.8% women) were included. Patients aged 15–49 years living in rural areas showed a 5% higher risk of ischemic stroke (IRR 1.05 [99% CI 0.98–1.13]) compared with patients in urbanized areas, whereas for persons aged 50 years and older, this risk was decreased by 3% (IRR 0.97 [99% CI 0.95–0.98]). For patients aged 15–39 years, this risk was 20% higher (IRR 1.20 [99% CI 1.05–1.37]), and for patients aged 40–49 years, the risk did not differ (IRR 1.01 [99% CI 0.93–1.09]). The rural-urban disparities in all age groups remained similar when stratified for nSES.

**Discussion:**

The incidence of ischemic stroke is higher among persons aged 15–49 years living in rural areas compared with urban areas, which was driven by a risk-increase in patients 15–39 years. This was reversed among persons aged 50 years and older. Our findings were not fully explained by differences in nSES. This suggests that different age-specific predictors might play a role in rural-urban disparities in ischemic stroke incidence.

## Introduction

Stroke is the second ranked cause of death and important cause of disability globally, accompanied by high direct and indirect socioeconomic burden.^1^ We need to identify novel stroke risk factors, which could be a target for preventive strategies. Apart from the well-known vascular risk factors of stroke, rural-urban disparities in the incidence of ischemic stroke receive increasing attention with studies reporting a higher incidence in rural areas compared with urban areas.^2-8^ Studies found a higher prevalence of well-known vascular risk factors in rural areas, but this was partially to completely attributable to a low socioeconomic status (SES).^2^ However, it is unknown if this holds true for all ages.

The incidence of ischemic stroke in young adults is increasing, while a decrease in patients aged 50 years or older has been reported.^9,10^ Although some age-specific risk factors and causes of stroke in young individuals have been identified, a large proportion of up to 30% remain without a clear cause. Identifying possible rural-urban disparities in the incidence of ischemic stroke in the young could be a stepping stone to unravel yet unknown risk factors.

We therefore investigated the age-specific incidence of ischemic stroke in rural and urbanized areas within the Netherlands, and if these findings could be explained by neighborhood SES (nSES).

## Methods

### Participants and Study Design

Patients were included in this study through linkage of the Dutch Hospital Discharge Register, the Population Register, and the National Cause of Death Register. Data are hosted by Statistics Netherlands and were available from 1995 to 2018. We included patients aged 15 years or older, with a first-ever ischemic stroke between January 1, 1998, and December 31, 2018, ensuring at least 3 years of medical history for all included patients to reliably select only a first-ever and a not recurrent event. Until 2012, these registers were linked through a combination of date of birth, sex, and postal code, resulting in approximately 86% linkage of Dutch citizens based on a unique combination of these factors.^11^ After 2012, Dutch citizens were linked based on their unique citizen's service number, meaning a 100% linkage between the registers. Details of these linkage procedures have been described in detail before.^12^

First-ever ischemic stroke was defined as the first hospitalization for ischemic stroke or stroke not otherwise specified, either with a primary or secondary discharge diagnosis, through the *International Classification of Disease, Ninth Revision* (*ICD-9*), and the *International Statistical Classification of Diseases and Related Health Problems, Tenth Revision* (*ICD-10*) codes. The validity of *ICD-9* and *ICD-10* codes has been previously assessed and found to be adequate.^10,13^ Stroke not otherwise specified was found to be ischemic strokes in most cases (80%).^10^ Therefore, we included this code in our study. We additionally linked our cohort of patients with the System of Social Statistical Datasets, also hosted by Statistics Netherlands, to determine the municipality of the patients' home address at date of stroke. Municipality division from 2018 was used, amounting to 380 municipalities.

### Risk Factors

The degree of urbanization of each municipality on January 1, 2018, was defined as the number of addresses per km^2^ divided by the surface of that area and was retrieved from StatLine Statistics Netherlands.^14^ We identified 5 degrees of urbanization: rural (<500 addresses per km^2^), hardly urbanized (≥500–<1,000 addresses per km^2^), moderately urbanized (≥1,000–<1,500 addresses per km^2^), strongly urbanized (≥1,500–<2,500 addresses per km^2^), and extremely urbanized (≥2,500 addresses per km^2^).^15^

The mean SES for each municipality was determined by the SES-Prosperity, Education, and Work (SES-PEW) score on January 1, 2018, and was retrieved from StatLine Statistics Netherlands.^16^ This score is based on multiple-component analysis per household using welfare (income and wealth) in deciles, highest level of education completed of main breadwinner and partner, and recent labor participation of main breadwinner and partner.^17^ We divided the scores in 3 categories: low nSES (SES-PEW score <−0.1), middle nSES (SES-PEW score ≥−0.1 to <0.1), and high nSES (SES-PEW score ≥0.1).

We calculated the Charlson Comorbidity Index (CCI) score at time of stroke.^18^ This is a validated score based on the presence of 19 primary medical conditions. We categorized the CCI in 4 groups: a score of 0, 1, 2, and 3 or more.^19^

### Statistical Analyses

Age, calendar year, and sex-adjusted standardized incidence ratio's and corresponding 99% CIs based on Poisson distribution were calculated to investigate the incidence of first-ever ischemic stroke per municipality compared with the overall incidence of the general population in the Netherlands. We used age-specific, year-specific, and sex-specific population estimates per municipality as reference of the general population, retrieved from Statline Statistics Netherlands.^20^ The expected number of cases was calculated by dividing the observed number of cases within the general population by the general population-size, multiplied by the population-size of the municipality. If a municipality had less than 10 observed cases in total, it was merged with an adjacent municipality to adhere to the privacy legislations of Statistics Netherlands. We stratified by age (15–49 years and ≥50 years).

We calculated the urbanization grade-specific incidence rate (IR) per 100,000 person-years, standardized for age and sex. To investigate the difference in incidence between urbanization grades, we calculated the incidence rate ratio (IRR) and corresponding 99% CI. We stratified for age groups (15–49 years and ≥50 years), sex, and nSES. We additionally stratified the young adult age group in 2 strata: 15–39 years and 40–49 years, given the different distribution of causes of stroke across these age groups.^21^ Significance between rural-urban disparities was defined as confidence intervals that do not cross the value of 1. To compare the risk between women and men, nonoverlapping confidence intervals were considered statistically significant.

### Standard Protocol Approvals, Registrations, and Patient Consents

This study was reported according to the Strengthening the Reporting of Observational Studies in Epidemiology guidelines. This study followed the guidelines of the Medical Ethical Committee region East-Netherlands. No ethical approval or patient consent was required according to the use of anonymous data and the ethical requirements from the local research committee.

### Data Availability

Results based on calculations in this study using nonpublic microdata from Statistics Netherlands. Under certain conditions, these microdata are accessible for statistical and scientific research. For further information: microdata@cbs.nl.

## Results

**Figure 1:**
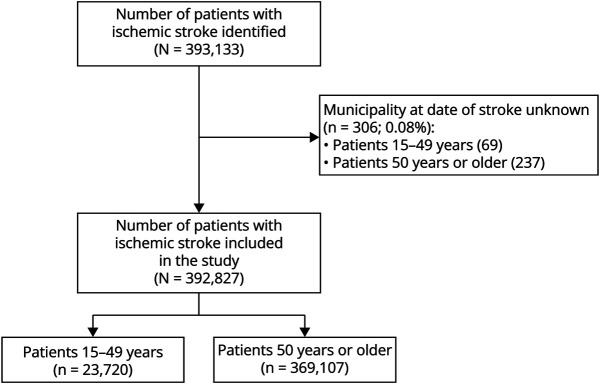
Flowchart of Included Patients With Ischemic Stroke

We identified 393,133 patients aged 15 years or older with a first-ever ischemic stroke between 1998 and 2018. The municipality at date of stroke was unknown for 306 (0.1%) patients, who were excluded from this study, resulting in a sample size of 392,827 patients (Figure 1). Of those, 23,720 (6.0%) patients were between 15 and 49 years of age at time of stroke (median age: 44.7 years [interquartile range (IQR) 40.6–48.8], 51.6% female) and 369,107 (94.0%) were 50 years or older (median age: 76.7 years [IQR 68.8–84.7], 50.8% female) (Table 1).

**Table 1:** Characteristics Included Patients

	Patients aged 15–49 y	Patients aged ≥50 y	Patients aged 15–39 y	Patients aged 40–49 y
Included patients, N	23,720	369,107	6,254	17,466
Age, y, median (IQR)	44.7 (40.6–48.8)	76.7 (68.8–84.7)	34.7 (30.7–38.7)	46.5 (44.3–48.7)
Women, n (%)	12,247 (51.6)	187,671 (50.8)	3,575 (57.2)	8,672 (49.7)
Charlson Comorbidity Index score, n (%)				
0	19,987 (84.3)	231,196 (62.6)	5,426 (86.8)	14,561 (83.4)
1	2,050 (8.6)	59,787 (16.2)	468 (7.5)	1,582 (9.1)
2	871 (3.7)	38,086 (10.3)	198 (3.2)	673 (3.9)
≥3	812 (3.4)	40,038 (10.8)	162 (2.6)	650 (3.7)
Period of first-ever stroke, n (%)				
1998–2008	10,702 (45.1)	159,854 (43.3)	3,043 (48.7)	7,659 (43.9)
2009–2018	13,018 (54.9)	209,253 (56.7)	3,211 (51.3)	9,807 (56.1)
Degree of urbanization of municipality at date of stroke, n (%)				
Rural	1,995 (8.4)	32,763 (8.9)	481 (7.7)	1,514 (8.7)
Hardly urbanized	5,060 (21.3)	83,513 (22.6)	1,320 (21.1)	3,740 (21.4)
Moderately urbanized	3,763 (15.9)	59,095 (16.0)	993 (15.9)	2,770 (15.9)
Strongly urbanized	7,497 (31.6)	111,612 (30.2)	1,959 (31.3)	5,538 (31.7)
Extremely urbanized	5,405 (22.8)	82,124 (22.5)	1,501 (24.0)	3,904 (22.4)
Mean SES of municipality at date of stroke, n (%)				
Low nSES	7,371 (31.1)	110,153 (29.8)	1,957 (31.3)	5,414 (31.0)
Middle nSES	10,320 (43.5)	157,412 (42.6)	2,747 (43.9)	7,573 (43.4)
High nSES	6,029 (25.4)	101,542 (27.5)	1,550 (24.8)	4,479 (25.6)

Abbreviations: IQR = interquartile range; nSES = neighborhood socioeconomic status.

A large portion of the patients were living in a strongly urbanized municipality at time of stroke (31.6% of the patients aged 15–49 years and 30.2% of the patients aged 50 years and older), and the least patients lived in a rural municipality (8.4% of the patients aged 15–49 years and 8.9% of the patients aged 50 years and older), which was similar to the distribution in the general population (Table 2).

**Table 2:** Characteristics of the 380 Municipalities in the Netherlands in 2018

	Extremely urbanized^a^	Strongly urbanized^a^	Moderately urbanized^a^	Hardly urbanized^a^	Rural^a^
Municipalities, N	19	74	78	135	74
Population, N	4,130,059	5,217,879	2,737,930	3,721,721	1,373,495
Women, n (%)	2,084,752 (50.5)	2,644,389 (50.7)	1,381,091 (50.4)	1,862,057 (50.0)	681,754 (49.6)
Age, n (%)					
<15 y	640,968 (15.5)	841,874 (16.1)	452,176 (16.5)	587,896 (15.8)	214,948 (15.7)
15–49 y	2,123,527 (51.4)	2,320,948 (44.5)	1,134,338 (41.4)	1,488,246 (34.0)	541,191 (39.4)
≥50 y	1,365,564 (33.1)	2,055,057 (39.4)	1,151,416 (42.1)	1,645,579 (44.2)	617,356 (45.0)
Address density, per km^2^, mean (SD)	3,293.42 (892.2)	1,852.19 (265.0)	1,241.28 (151.7)	719.84 (133.3)	367.82 (92.0)
Mean SES of municipality, n (%)					
Low nSES^b^	10 (52.6)	14 (18.9)	7 (9.0)	9 (6.7)	2 (2.7)
Middle nSES^b^	9 (47.4)	41 (55.4)	27 (34.6)	51 (37.8)	33 (44.6)
High nSES^b^	0 (0)	19 (25.7)	44 (56.4)	75 (55.6)	39 (52.7)
Distance to GP, km, mean (SD)	0.66 (0.1)	0.86 (0.2)	1.04 (0.3)	1.25 (0.3)	1.48 (0.5)
Distance to hospital, km, mean (SD)	2.61 (0.6)	3.27 (1.4)	4.21 (2.2)	7.34 (3.8)	12.89 (9.2)

Abbreviations: GP = general practitioner; nSES = neighborhood socioeconomic status.

a Degree of urbanization: rural <500 addresses per km^2^, hardly urbanized ≥500–<1,000 addresses per km^2^, moderately urbanized ≥1,000–<1,500 addresses per km^2^, strongly urbanized ≥1,500–<2,500 addresses per km^2^, and extremely urbanized ≥2,500 addresses per km^2^.

b SES-PEW (Prosperity, Education, and Work) score. Low nSES, SES-PEW score <−0.1; middle nSES, SES-PEW score ≥−0.1 to <0.1; and high nSES, SES-PEW score ≥0.1.

### Incidence of Ischemic Stroke by Urbanization Grade and Age

**Figure 2:**
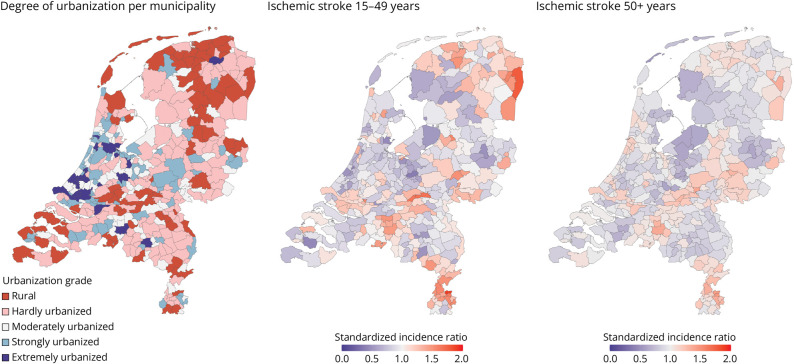
Standardized Incidence Ratio of Ischemic Stroke Per Municipality in the Netherlands Per municipality in the Netherlands: the degree of urbanization (left), standardized incidence ratio of ischemic stroke between 15 and 49 years (middle), and standardized incidence ratio of ischemic stroke at 50 years or older (right). Nine municipalities were merged for patients between 15 and 49 years old because of disclosure risk. Degree of urbanization: rural <500 addresses per km^2^, hardly urbanized ≥500–<1,000 addresses per km^2^, moderately urbanized ≥1,000–<1,500 addresses per km^2^, strongly urbanized ≥1,500–<2,500 addresses per km^2^, and extremely urbanized ≥2,500 addresses per km^2^.

The incidence of ischemic stroke per municipality is depicted in Figure 2. The risk of ischemic stroke in young adults aged 15–49 years was higher in rural areas compared with extremely urban areas, although not significant (IR 14.9 vs 14.1; IRR 1.05 [99% CI 0.98–1.13]), Table 3. Persons aged 50 years or older had a statistically significant lower IR per 100,000 person years in rural areas compared with extremely urbanized areas (IR 308.98 vs 319.87; IRR 0.97 [99% CI 0.95–0.98]). Young women in rural areas had a significant higher risk of ischemic stroke, compared with young women in extremely urbanized areas (IR 15.9 vs 14.3 IRR 1.11 [99% CI 1.01–1.22]). Young women in rural areas did not have a significant higher risk compared with young men in rural areas (men: IRR 0.99 [99% CI 0.90–1.09]), which was similar in persons 50 years or older (women: IRR 0.98 [99% CI 0.96–1.00] and men: IRR 0.95 [99% CI 0.93–0.97]).

**Table 3:** Urbanization-Specific Incidence Rate Per 100,000 Person-Years, Standardized for Age and Sex, and Incidence Rate Ratios of Ischemic Stroke

	Aged 15–49 y					Aged 50 y or older				
	Population size	Observed events	Expected events	Incidence rate^a^	Incidence rate ratio (99% CI)	Population size	Observed events	Expected events	Incidence rate^a^	Incidence rate ratio (99% CI)
Total										
Rural	12,593,361	1,995	1,814	14.9	1.05 (0.98–1.13)	10,778,435	32,763	33,238	309.0	0.97 (0.95–0.98)
Hardly urbanized	34,133,997	5,060	4,917	14.0	0.99 (0.94–1.04)	28,630,900	83,513	88,293	297.1	0.93 (0.92–0.94)
Moderately urbanized	25,071,976	3,763	3,611	14.3	1.01 (0.96–1.07)	19,723,793	59,095	60,825	306.9	0.96 (0.95–0.97)
Strongly urbanized	50,334,983	7,497	7,251	15.0	1.06 (1.01–1.11)	35,754,567	111,612	110,261	313.5	0.98 (0.97–0.99)
Extremely urbanized	42,517,151	5,405	6,125	14.1	1 (reference)	24,803,088	82,124	76,488	319.9	1 (reference)
Women										
Rural	6,124,144	1,034	921	15.9	1.11 (1.01–1.22)	5,543,472	16,024	16,458	298.8	0.98 (0.96–1.00)
Hardly urbanized	16,707,093	2,567	2,512	14.49	1.02 (0.95–1.09)	14,895,272	41,758	44,223	289.8	0.95 (0.94–0.97)
Moderately urbanized	12,396,000	2,028	1,864	15.6	1.09 (1.01–1.18)	10,383,885	29,723	30,829	296.9	0.97 (0.96–0.99)
Strongly urbanized	25,079,493	3,931	3,772	15.75	1.11 (1.04–1.18)	19,051,882	57,075	56,564	301.4	1.00 (0.97–1.01)
Extremely urbanized	21,117,648	2,687	3,176	14.25	1 (reference)	13,336,282	43,091	39,595	304.6	1 (reference)
Men										
Rural	6,469,217	961	891	13.9	0.99 (0.90–1.09)	5,234,963	16,739	16,816	320.4	0.95 (0.93–0.97)
Hardly urbanized	17,426,904	2,493	2,402	13.6	0.97 (0.90–1.04)	13,735,628	41,755	44,124	305.2	0.91 (0.89–0.92)
Moderately urbanized	12,675,976	1,735	1,747	13.1	0.93 (0.86–1.01)	9,339,908	29,372	30,003	318.1	0.94 (0.93–0.96)
Strongly urbanized	25,255,490	3,566	3,481	14.2	1.01 (0.95–1.08)	16,702,685	54,537	53,655	327.0	0.97 (0.95–0.99)
Extremely urbanized	21,399,503	2,718	2,949	14.0	1 (reference)	11,466,806	39,033	36,835	337.0	1 (reference)
Low nSES										
Rural	168,217	31	24	15.1	1.13 (0.71–1.80)	135,458	369	458	287.3	0.86 (0.75–0.98)
Hardly urbanized	3,147,837	641	452	17.8	1.34 (1.20–1.49)	2,807,323	9,627	9,500	341.7	1.02 (0.99–1.05)
Moderately urbanized	2,899,366	526	416	16.0	1.20 (1.07–1.36)	2,355,106	8,065	7,970	363.6	1.08 (1.05–1 12)
Strongly urbanized	13,260,140	2,086	1,904	15.5	1.16 (1.08–1.24)	9,198,095	31,149	31,128	342.9	1.02 (1.00–1.04)
Extremely urbanized	31,847,974	4,087	4,573	13.3	1 (reference)	18,053,300	60,943	61,095	335.3	1 (reference)
Middle nSES										
Rural	5,777,005	998	870	16.5	1.23 (1.10–1.37)	5,089,723	15,690	15,519	307.6	1.02 (0.99–1.05)
Hardly urbanized	14,537,294	2,155	2,191	14.3	1.06 (1.97–1.16)	12,259,820	35,423	37,382	292.5	0.97 (0.95–0.99)
Moderately urbanized	9,636,435	1,594	1,452	16.3	1.21 (1.10–1.33)	7,660,671	23,906	23,358	314.2	1.04 (1.02–1.07)
Strongly urbanized	27,848,339	4,255	4,197	15.4	1.15 (1.06–1.24)	19,864,018	61,212	60,569	311.9	1.04 (1.01–1.06)
Extremely urbanized	10,669,177	1,318	1,608	13.4	1 (reference)	6,749,788	21,181	20,581	301.1	1 (reference)
High nSES										
Rural	6,648,139	966	893	14.4	1.13 (1.01–1.30)	5,553,254	16,704	15,876	303.3	1.08 (1.05–1.11)
Hardly urbanized	16,448,866	2,264	2,210	13.7	1.08 (0.98–1.18)	13,563,757	38,463	38,777	286.0	1.02 (1.00–1.04)
Moderately urbanized	12,536,175	1,643	1,684	13.1	1.02 (0.93–1.13)	9,708,016	27,124	27,754	282.0	1.00 (0.98–1.03)
Strongly urbanized	9,226,504	1,156	1,240	12.8	1 (reference)	6,692,454	19,251	19,133	280.9	1 (reference)
Extremely urbanized^b^	NA	NA	NA	NA	NA	NA	NA	NA	NA	NA

Abbreviations: NA = not applicable; nSES = neighborhood socioeconomic status.

Rural was defined as <500 addresses per km^2^, hardly urbanized ≥500–<1,000 addresses per km^2^, moderately urbanized ≥1,000–<1,500 addresses per km^2^, strongly urbanized ≥1,500–<2,500 addresses per km^2^, and extremely urbanized ≥2,500 addresses per km^2^. SES was defined by the SES-PEW (Prosperity, Education, and Work) score. Low nSES, SES-PEW <−0.1; middle nSES, SES-PEW ≥−0.1 to <0.1; and high nSES, SES-PEW ≥0.1.

a Incidence rates were standardized for age and sex.

b There were no municipalities with an extremely urbanized grade and a mean high nSES.

When the younger age group is further stratified, the risk-increase in rural areas was significantly higher for the youngest group of 15–39 years (IR 5.9 vs 4.9; IRR 1.20 [95% CI 1.05–1.37]), but not for people aged 40–49 years (IR 34.5 vs 34.3; IRR 1.03 [95% CI 0.97–1.08]) (Table 4). In both age strata, this risk was more pronounced in women (for 15–39 years, women: IRR 1.30 [99% CI 1.09–1.55]; men: IRR 1.07 [99% CI 0.86–1.32], and for 40–49 years, women: IRR 1.04 [99% CI 0.93–1.17]; men: 0.97 [99% CI 0.87–1.09]). The patterns for all age groups were similar when we compared the incidence rates of extremely urbanized areas with the other levels of rurality (i.e., strongly, moderately, and hardly urbanized areas).

**Table 4:** Urbanization-Specific Incidence Rate Per 100,000 Person-Years, Standardized for Age and Sex, and Incidence Rate Ratios of Ischemic Stroke Stratified for Age Strata 15–39 Years and 40–49 Years

	Aged 15–39 y					Aged 40–49 y				
	Population size	Observed events	Expected events	Incidence rate^a^	Incidence rate ratio (99% CI)	Population size	Observed events	Expected events	Incidence rate^a^	Incidence rate ratio (99% CI)
Total										
Rural	8,231,870	481	456	5.9	1.20 (1.05–1.37)	4,361,491	1,514	1,470	34.5	1.01 (0.93–1.09)
Hardly urbanized	22,403,399	1,320	1,241	5.9	1.20 (1.09–1.32)	11,730,598	3,740	3,955	31.7	0.93 (0.87–0.98)
Moderately urbanized	16,610,683	993	920	5.9	1.21 (1.09–1.34)	8,461,293	2,770	2,853	32.6	0.95 (0.89–1.02)
Strongly urbanized	34,593,905	1,959	1,917	5.6	1.16 (1.06–1.26)	15,741,078	5,538	5,307	35.2	1.03 (0.97–1.08)
Extremely urbanized	31,014,016	1,501	1,718	4.9	1 (reference)	11,503,135	3,904	3,878	34.3	1 (reference)
Women										
Rural	3,973,450	288	254	7.3	1.30 (1.09–1.55)	2,150,694	746	726	34.6	1.04 (0.93–1.17)
Hardly urbanized	10,892,715	730	698	6.7	1.20 (1.05–1.36)	5,814,378	1,837	1,963	31.5	0.95 (0.87–1.04)
Moderately urbanized	8,166,515	599	523	7.2	1.30 (1.13–1.49)	4,229,485	1,429	1,428	33.7	1.02 (0.93–1.12)
Strongly urbanized	17,220,438	1,118	1,104	6.5	1.17 (1.04–1.31)	7,859,055	2,813	2,654	35.8	1.08 (1.00–1.17)
Extremely urbanized	15,496,067	840	993	5.6	1 (reference)	5,621,581	1,847	1,898	33.1	1 (reference)
Men										
Rural	4,258,420	193	199	4.5	1.07 (0.86–1.32)	2,210,797	768	744	34.5	0.97 (0.87–1.09)
Hardly urbanized	11,510,684	590	540	5.1	1.21 (1.04–1.40)	5,916,220	1,903	1,991	32.0	0.90 (0.83–0.98)
Moderately urbanized	8,444,168	394	396	4.6	1.09 (0.93–1.28)	4,231,808	1,341	1,424	31.6	0.89 (0.81–0.98)
Strongly urbanized	17,373,467	841	815	4.8	1.14 (1.00–1.30)	7,882,023	2,725	2,653	34.6	0.98 (0.91–1.05)
Extremely urbanized	15,517,949	661	728	4.3	1 (reference)	5,881,554	2,057	1,980	35.4	1 (reference)

Rural was defined as <500 addresses per km^2^, hardly urbanized ≥500–<1,000 addresses per km^2^, moderately urbanized ≥1,000–<1,500 addresses per km^2^, strongly urbanized ≥1,500–<2,500 addresses per km^2^, and extremely urbanized ≥2,500 addresses per km^2^.

a Incidence rates were standardized for age and sex.

### Incidence of Ischemic Stroke by Urbanization Grade, Stratified by nSES

When stratified by nSES, for adults aged 15–49 years, the risk-increase in rural areas compared with extremely urbanized areas remained higher in all nSES categories. The highest risk-increase of stroke at young age for rural compared with extremely urbanized areas was observed in middle nSES municipalities (IR 16.5 vs 13.4; IRR 1.23 [95% CI 1.10–1.37]), compared with low nSES (IR 15.1 vs 13.3; IRR 1.13 [95% CI 0.71–1.80]), and high nSES (IR 14.4 vs 12.8; IRR 1.13 [95% CI 1.01–1.30]).

The IR of stroke in persons aged 50 years or older was lower in rural areas compared with extremely urbanized areas for low nSES (IR 287.3 vs 335.3, IRR 0.86 [95% CI 0.75–0.98]). For middle and high nSES, the incidence was higher (middle: IR 307.6 vs 301.1, IRR 1.02 [95% CI 0.99–1.05]; high: IR 303.3 vs 280.9, IRR 1.08 [95% CI 1.05–1.11]). There were no municipalities with an extremely urbanized grade and a mean high nSES; thus, for high nSES municipalities, rural and strongly urbanized areas were compared.

## Discussion

We found that young adults living in rural areas have a higher risk of ischemic stroke compared with young adults in extremely urbanized areas, which was driven by the 20% risk-increase for persons 15–39 years old. By contrast, persons older than 50 years living in rural areas had a 3% lower risk of ischemic stroke compared with persons in extremely urbanized areas. Women showed a higher risk in rural areas, compared with men in all age groups. The rural-urban disparities in both age groups remained similar when stratified by nSES, although the effect of urbanicity was more pronounced in municipalities with a low nSES vs municipalities with a high nSES.

Previous studies similarly found a higher stroke incidence in rural areas, but did not investigate age-specific incidences.^2-6^ Our results suggest an age-dependent effect of urbanicity on stroke incidence. Although the absolute incidence of stroke is much lower in younger compared with older age groups, the 20% relative increase in rural areas among young people between 15 and 39 years is of clinical importance because it may provide leads to unravel the cause of stroke in young adults, which remains unknown in approximately one-third of young patients. Preventing stroke at young age is key, given the long-term personal and socioeconomic impact.

A possible explanation for an increased risk for young adults in rural areas could be differences in vascular risk factors and lifestyle that may increase the risk of ischemic stroke at a young age. A previous study from the United States, including persons aged 45 years or older, reported a 25% higher odds of hypertension and 15% higher odds of diabetes in rural areas.^2^ This was in line with a Swedish study including persons between 25 and 75 years that reported a higher prevalence of low education, obesity, and hypercholesteremia in rural areas, compared with urbanized areas.^22^ Findings from a Chinese study showed that family history of stroke predicted a stroke in rural residents.^23^ This could be explained by the fact that families with a hereditary high risk of stroke, possibly occurring at a younger age, stay in rural areas. In addition, the odds of substance use (e.g., excessive alcohol use, tobacco use, cannabis use, and illicit drug use) was 36% higher in young American adults in rural areas, compared with their peers in urbanized areas.^24,25^ In the Netherlands, it has been reported that youths in rural areas more often engage in excessive alcohol use. A second contributing factor could be the higher vaccination grades in urban areas.^26^ A previous study found a 10%–15% lower risk of ischemic stroke after influenza vaccination.^27^ The role of inflammation is also increasingly recognized in ischemic stroke at young age.^28^ Finally, pesticide exposure in rural areas has been associated with increased risk of atherosclerosis and other cardiovascular diseases, which might result in vascular reactivity and proinflammatory processes that result in vascular disease already earlier in life compared with persons without regular exposure to these pesticides.^29,30^ Future clinical studies should investigate whether these risk factors can explain the rural-urban disparities in ischemic stroke at young age.

By contrast, we report a lower risk of ischemic stroke for persons aged 50 years or older living in rural areas compared with extremely urbanized areas, which is a novel finding. One reason for the different findings in our work compared with previous studies could be methodologic differences. First, we used a different definition of rurality. Studies from the United States and Canada used definitions based on location and number of residents at the census tract level (statistical subdivisions within a county with, respectively, <2,500 residents per subdivision and <10,000 residents per subdivision not in a commuting area of a metropolitan town, respectively),^3,31^ while we defined rurality according to the address density (<500 addresses per km^2^) of a municipality, hampering comparison of the results. Second, we included only first-ever strokes, meaning that patients who already had a stroke at young age (and who are at a higher risk for a recurrent stroke) are not part of the patients aged 50 years or older cohort in our study. By not including recurrent strokes, the high risk in rural areas of overall stroke in the older population of our study may be underestimated. Last, the incidence of ischemic stroke at age 50 years or older might be diminished because of selective survival bias, meaning persons with poorer cardiovascular health living in rural areas might have died before reaching the age of 50.

A possible explanation for the contrasting rural-urban disparities in persons 50 years or older can be that previous studies were conducted in larger countries in which the distance to primary stroke care in rural areas is larger. This makes it more demanding for persons to visit a general practitioner for regular health check-ups that may potentially lead to underdiagnosis and treatment of vascular risk factors. This results in insufficient risk factor management, which becomes of more importance with increasing age. This is less of a problem in the densely populated Netherlands because distances to a general practitioner and hospital throughout all areas are small. A second reason could be long-term cumulative exposure to ambient air pollution in urban areas, which is associated with signs of inflammation and coagulation, and for which the older population is more susceptible.^32^

When stratified by nSES, the effects of urbanicity on stroke incidence in both age groups remained, though were most pronounced in municipalities with a mean low nSES. In current literature, a low SES has been associated with a higher incidence of cardiovascular risk factors.^33,34^ The REGARDS study found that the effect of hypertension and diabetes on the increased risk of stroke in rural areas was partially to completely attributable to a lower nSES.^2^ This could possibly be explained by the fact that patients in a municipality with a higher nSES have better health-awareness and better access to primary health care.^33,35^ However, in our study, nSES does not seem to completely explain the differences between rural and urban stroke incidence, indicating that other clinical and environmental risk factors might be of importance. Unfortunately, due to the privacy legislation of statistics Netherlands, we could not investigate the risk of the younger age strata (15–39 years) per nSES. In addition, we found more pronounced rural-urban disparities in women compared to men. We can hypothesize that women might be more susceptible for certain hormonal or genetical risk factors for ischemic stroke that play a role in rural-urban disparities.^36^ However, future research should determine which women-specific risk factors are of importance.

This study provides leads for future clinical research to investigate age-specific effects on the rural-urban disparities in ischemic stroke incidence. It implies that different factors are associated with the effect of urbanicity on stroke incidence for young adults compared with persons 40 years and older are at play. Factors that might be of interest are risk factors such as excessive alcohol use, illicit drug use, family history of stroke, ambient air pollution, health-awareness, systemic inflammation, pesticide exposure, women-specific hormonal or genetical factors, and clinical characteristics of stroke. Future studies are needed to evaluate these risk factors on urbanicity-specific stroke incidence to unravel possible underlying mechanisms of stroke at young age. In addition, our study suggests that current primary stroke prevention, such as increasing awareness, might be beneficial for young adults in rural areas.

This study has some important strengths. First, we investigated rural-urban disparities for young adults and persons 50 years or older separately in a large registry-based cohort study. Second, we stratified for mean nSES per municipality. Third, our results are most likely not explained by limited access to primary stroke care in rural areas because distances to a general practitioner or hospital in the Netherlands are small. There are however also some limitations. First, we did not have individual patient data about stroke characteristics, presence of cardiovascular risk factors, ethnicity, or SES; thus, we could not investigate the role of these risk factors in rural-urban disparities. Second, we included patients with stroke “not otherwise specified,” which means that some of these patients might have had an intracerebral hemorrhage instead of an ischemic stroke. However, a validation study found that 80% of all strokes not otherwise specified were ischemic strokes, making this risk of misdiagnosis limited. Third, there might be heterogeneity in the address density across a municipality and in number of residents per address across the levels of rurality. This could have resulted in an overestimation or underestimation of the reported urbanization-specific risk. Last, we have no medical information available before 1995, meaning we could have included some recurrent strokes. To decrease the risk of overestimation of first-ever strokes, we included strokes from 1998 onward because recurrent strokes mostly occur in the first few years after a primary stroke.

In conclusion, we found clear rural-urban disparities in the incidence of ischemic stroke at young age, which was inverted for persons 50 years or older. This suggests that different age-specific factors might play a role in rural-urban disparities in ischemic stroke incidence. Our study promotes future research to investigate possible primary prevention of ischemic stroke focused on young adults in rural areas.

## Appendix Authors

**Table:**

Name	Location	Contribution
Esmée Verburgt, MSc	Department of Neurology, Radboud University Medical Center, Donders Institute for Brain, Cognition and Behavior, Nijmegen, the Netherlands	Drafting/revision of the manuscript for content, including medical writing for content; study concept or design; analysis or interpretation of data
Jamie I. Verhoeven, MD	Department of Neurology, Radboud University Medical Center, Donders Institute for Brain, Cognition and Behavior, Nijmegen, the Netherlands	Drafting/revision of the manuscript for content, including medical writing for content; study concept or design; analysis or interpretation of data
Nina A. Hilkens, MD, PhD	Department of Neurology, Radboud University Medical Center, Donders Institute for Brain, Cognition and Behavior, Nijmegen, the Netherlands	Drafting/revision of the manuscript for content, including medical writing for content; analysis or interpretation of data
Ilonca Vaartjes, PhD	Department of Epidemiology, Julius Center for Health Sciences and Primary Care, University Medical Center Utrecht, the Netherlands	Drafting/revision of the manuscript for content, including medical writing for content
Frank-Erik De Leeuw, MD, PhD	Department of Neurology, Radboud University Medical Center, Donders Institute for Brain, Cognition and Behavior, Nijmegen, the Netherlands	Drafting/revision of the manuscript for content, including medical writing for content; analysis or interpretation of data

## Glossary

CCI: Charlson Comorbidity Index
*ICD-9*: *International Classification of Diseases, Ninth Revision*
*ICD-10*: *International Statistical Classification of Diseases and Related Health Problems, Tenth Revision*
IQR: interquartile range
IR: incidence rate
IRR: incidence rate ratio
nSES: neighborhood SES
SES: socioeconomic status
SES-PEW: SES-Prosperity, Education, and Work
